# Nickel Oxide Nanoparticles Derived from Coordination Polymer of PVA and Aminobenzoic Acid Derivative: Synthesis, Characterization and Antimicrobial Activity

**DOI:** 10.3390/polym17030301

**Published:** 2025-01-23

**Authors:** Maged S. Al-Fakeh, Roaa O. Alsaedi, Maryam Aldoghaim, Ahmed B. M. Ibrahim, Ayman M. Mostafa

**Affiliations:** 1 Department of Chemistry, College of Science, Qassim University, Buraidah 51452, Saudi Arabia; roaa.alsaadii@gmail.com; 2 Department of Chemistry, College of Science, King Faisal University, Al-Ahsa 31982, Saudi Arabia; maldoghaim@kfu.edu.sa; 3 Chemistry Department, College of Science, Imam Mohammad Ibn Saud Islamic University (IMSIU), Riyadh 11623, Saudi Arabia; abibrahim@imamu.edu.sa; 4 Department of Physics, College of Science, Qassim University, Buraidah 51452, Saudi Arabia; a.mostafa@qu.edu.sa

**Keywords:** organometallics, metal oxide nanoparticles, kinetic, SEM, TEM, antimicrobial activity

## Abstract

This study focused on the synthesis, properties, and antibiological activity of NiO nanoparticles derived from polyvinyl alcohol (PVA) and aminobenzoic acid (P-ABA) derivatives by calcination method. The nanoparticles were synthesized using a simple, cost-effective method that involved the thermal decomposition of PVA and the incorporation of aminobenzoic acid. Characterization techniques such as X-ray diffraction (XRD), Kinetic analysis, and the thermal properties of nickel(II) metal complex in dynamic air were analyzed via TG and DTG. The kinetic analyses and thermodynamic parameters (∆H*, ∆G*, and ∆S*) for this compound were calculated by the Coats–Redfern and Horowitz–Metzger methods. The obtained kinetic parameters displayed the kinetic compensation effect. Electron microscopy (SEM and TEM) and (FT-IR) were employed to confirm the formation, morphology, and structural properties of the nanoparticles. The results indicated the successful synthesis of NiO nanoparticles with distinct crystalline phases and difference distributions. XRD confirmed that the resulting oxide was pure single-crystalline NiO nanoparticles. Scanning electron microscopy indicated that the crystallite size of nickel oxide nano-crystals was in the range of 26–36 nm. The magnetic moment was 2.59 B.M for Ni(II) complex. The antibiological activity of the synthesized nanoparticles was evaluated against bacterial strains, both Gram-positive and Gram-negative bacteria. The findings revealed significant antimicrobial properties, with the NiO nanoparticles demonstrating higher inhibitory effects against bacterial and fungal strains. This study highlights the potential of PVA and aminobenzoic acid derivatives as effective precursors for producing metal oxide nanoparticles with promising applications in antimicrobial treatments and materials science.

## 1. Introduction

**Figure 1:**
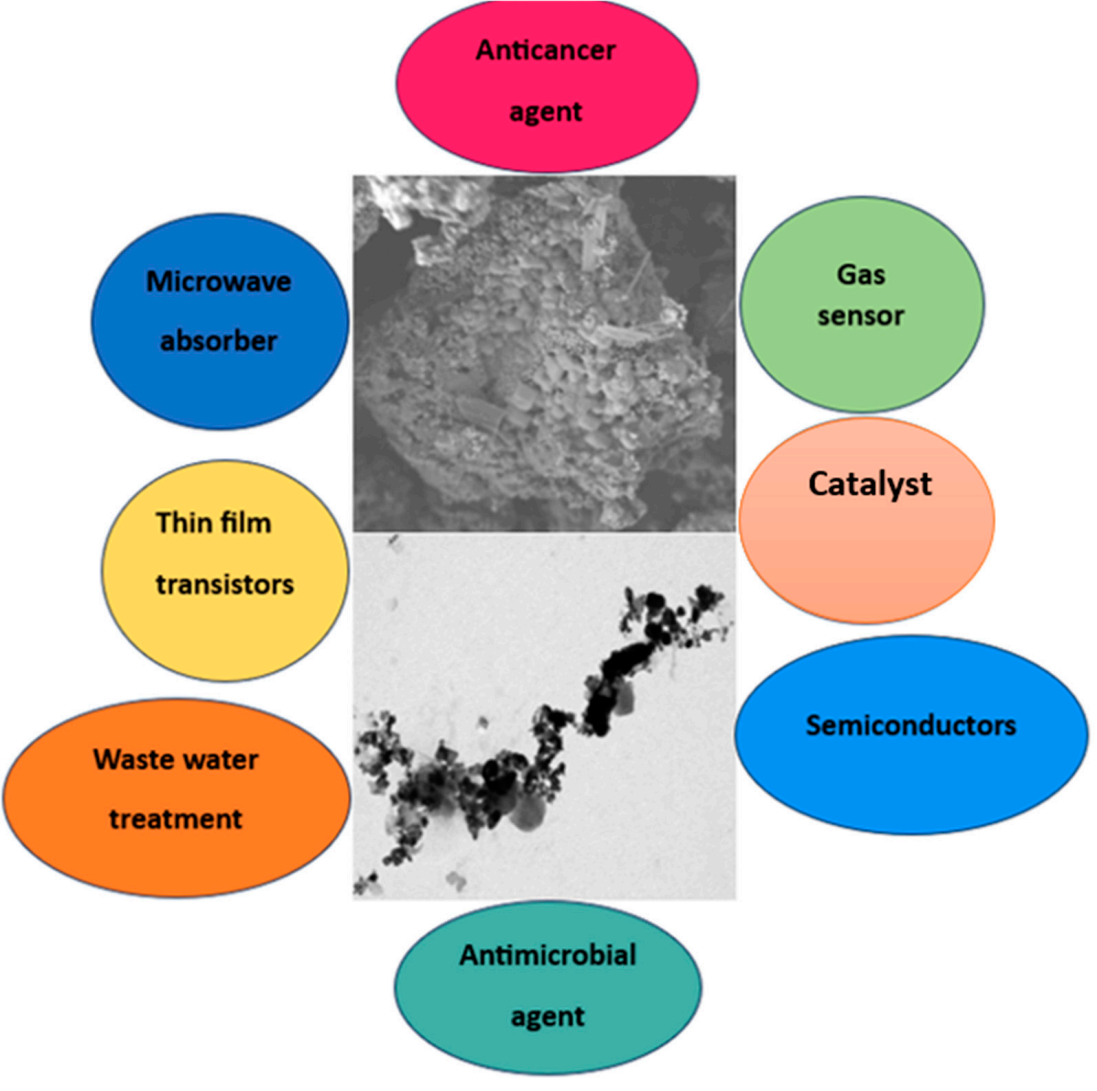
Nickel oxide nanoparticles with some applications.

In recent years, metal oxide nanoparticles have garnered significant attention in various fields because of their unique physical and chemical properties, such as reactivity, high surface area, and optical characteristics. These nanoparticles, typically composed of materials like titanium dioxide, copper oxide, zinc oxide, and nickel oxide, are utilized in applications ranging from catalysis, electronics, and environmental remediation to drug delivery and medical and pharmaceutical applications [1,2,3,4,5,6,7]. Their size, generally ranging from 1 to 100 nanometers, allows them to exhibit enhanced performance compared to their bulk counterparts, making them a focal point of research and innovation in nanotechnology [8,9]. Figure 1 illustrates the applications of NiO NPs. Nickel oxide NPs are the most frequently utilized photocatalyst for the destruction of various chemical compounds, bacteria, and viruses, as well as for water filtration [10,11]. Since the performance of photocatalysts is highly dependent on nanotechnology, where it rises as the size of NiO nanoparticles decreases. Nickel oxide nanoparticles have become increasingly important in “water treatment and removal of organic materials”. This is due to the fact that these materials have superior stability, transparency, availability, and nontoxicity, in addition to strong photocatalyst activity, which has proven to be superior to that of other photocatalysts in recent research [12,13,14,15,16].

Polyvinyl alcohol (PVA) is a synthetic polymer known for its water solubility and film-forming properties, making it useful in various applications, such as adhesives, coatings, and textiles [17,18,19,20]. Aminobenzoic acid, a derivative of benzoic acid, has important roles in organic synthesis and is often used in pharmaceuticals and as a UV-absorbing agent in sunscreens [21,22,23]. The combination of PVA and aminobenzoic acid can enhance the performance of materials, particularly in applications requiring both structural integrity and UV protection. In this article, the synthesis and properties of the Ni(II) compound-derived PVA and p-aminobenzoic acid ligands are described, and the resulting compound is used as a raw material for the synthesis of NiO nanoparticles, which is compared with previous studies, where these newly prepared oxide show better results and clear activity on selected bacteria and fungi that cause diseases, such as particularly those synthesized through microwave, sol-gel, or green methods in antibacterial applications. The effectiveness of these nanoparticles can be influenced by factors such as size, morphology, and synthesis method [24,25,26].

## 2. Materials and Methods

### 2.1. Materials

The chemicals were obtained commercially and used without otherwise purification. Polyvinyl alcohol (PVA), P-aminobenzoic acid, ethanol, and nickel(II) chloride hexahydrates (99%) were purchased from Sigma-Aldrich (St. Louis, MO, USA).

### 2.2. Preparation of [Ni(PVA)(P-ABA)(H_2_O)_3_]·H_2_O Complex

A sample of 2 g of PVA was dissolved in 100 mL of distilled water; the solution was stirred at 50 °C for 1 h, and then, a solution of “NaOH (0.001 M in 10 mL H_2_O)” was added to the above solution. When the mixture cooled, 6.42 g of NiCl_2_·6H_2_O was added with 15 mL distilled water by drops under stirring. After that, 3.70 g of P-ABA was dissolved in 25 mL ethanol and then added to the mixture; the stirring was continued for 3 h. A light green precipitate formed after cooling and then was separated by filtration.

### 2.3. Preparation of NiO Nanoparticles

The light green precipitate of Ni(II) complex was oven-dried at 25 °C, then directly calcined at 450 °C for 3 h.

### 2.4. Characterization Techniques

Elemental analyses (C, H, N) were performed using an Analyischer Function test Var. El Fab Nr. (11982027) elemental analyzer. The FT-IR spectra were acquired using a Thermo-Nicolet-6700 spectrophotometer between 400 and 4000 cm^−1^ “on a KBr disk”. With a “PC SHIMADZU UV-2102 UV-vis spectrophotometer (Shimadzu, Kyoto, Japan)”, the UV-vis spectrum was recorded in “1 cm quartz cuvettes over the wavelength range of 200–900 nm”. Magnetic moments of the fabricated complexes were measured at room temperature using a Sherwood Scientific Magnetic Susceptibility Balance from MSB-Auto. Melting points (mps) were determined on an automatic (SMP30) melting point apparatus. The conductance was determined using a “conductance meter (4310 JENWAY, Cole-Parmer Ltd., Vernon Hills, IL, USA)”. Thermogravimetric analysis was performed on a “Shimadzu Corporation 60H (Shimadzu, Kyoto, Japan)”, recording the thermogravimetric curves (TG, DTG, and DTA) of the investigated solid compounds at 40 mL min^−1^ in dynamic air; the temperature was raised from “20 °C to 750 °C at a rate of 10 °C min^−1^”. Crystallographic data for the structure of the compound were collected using an “XRD Model PW 1710 control unit Philips (Amsterdam, The Netherlands) with a Cu Kα (λ = 1.54180 Å) anode at 40 K.V 30 M.A Optics: Slit for automatic divergence”. The TREOR program was used to compute and apply the crystal lattice parameters. The morphology of the structures was observed by “SEM and TEM images that have been collected in a scanning electron microscope (Tecnai T12, FEI, Hillsboro, OR, USA) instrument. The microscope operated at 120 kV” for the transmission electron microscopy.

### 2.5. Biological Activity

In this study, the antimicrobial activities of metal oxide nanoparticles mentioned above were extracted and estimated by Dr. Rejo Joseph at Nitte University, Mangalore, India, “using five bacterial strains and three fungal strains. Three strains of Gram-positive cocci (staphylococcus aureus, staphylococcus epidermidis, and *Enterococcus faecalis*), two strains of Gram-negative bacilli (*Escherichia coli* and *Pseudomonas aeruginosa*), one stain of yeast-like fungi (*Candida albicans*), and two molds (*Aspergillus fumigatus* and *Aspergillus flavus*)” were evaluated.

## 3. Results

**Figure 2:**
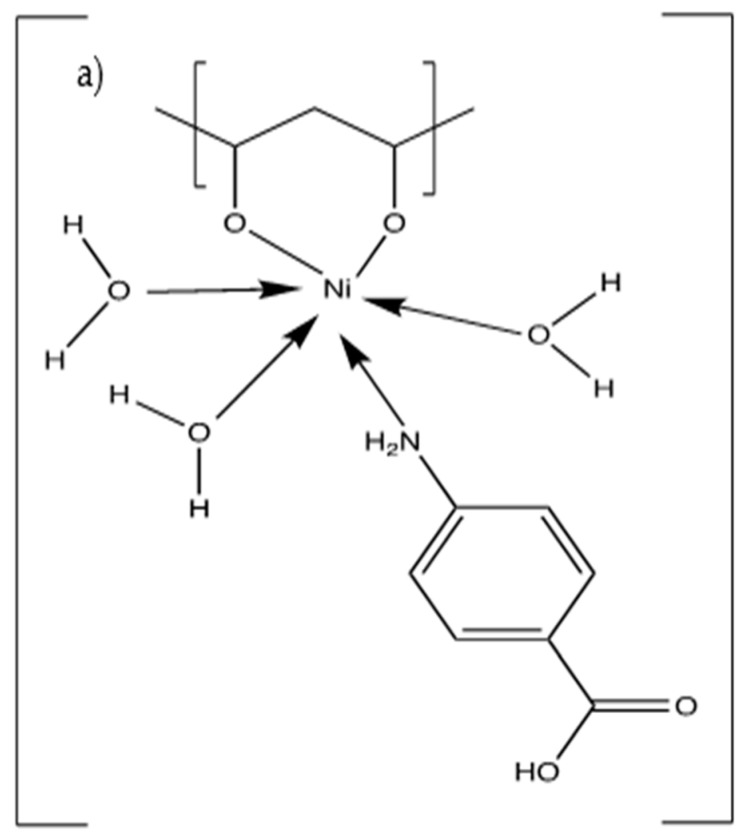

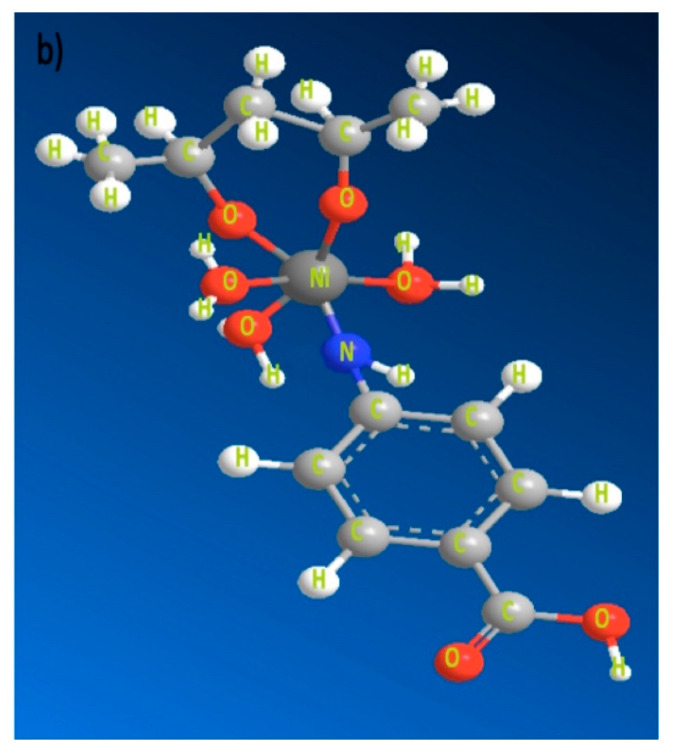
[Ni(PVA)(*P*-ABA)(H_2_O)_3_]·H_2_O complex. (**a**) Proposed structure; (**b**) coordination structure around Ni(II) complex.

**Scheme 1:**
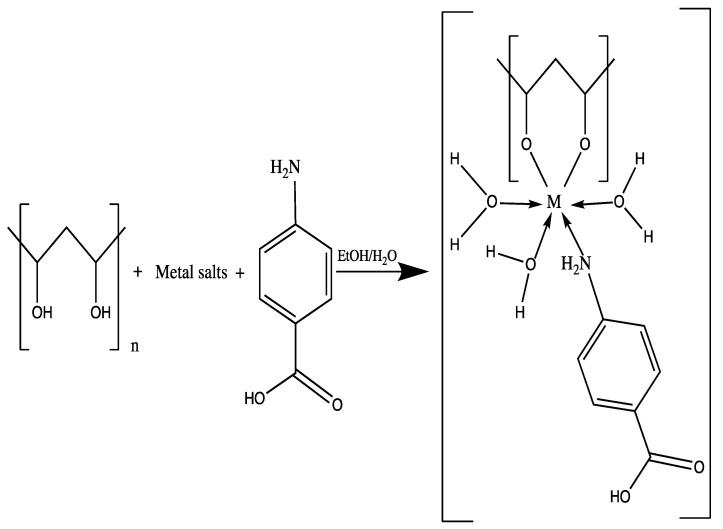
Synthesis of metal complex, where M = Ni(II).

The Ni(II) compound shows an octahedral position in Figure 2a, and the suggestion of coordination structure around nickel(II) is shown in Figure 2b. Synthesis of this complex undergoes Scheme 1. The structural information and crystals obtained were characterized through diverse techniques, which are discussed in greater detail in the following section. The elemental analysis is shown in Table 1.

### 3.1. FT-IR Spectroscopy

**Figure 3:**
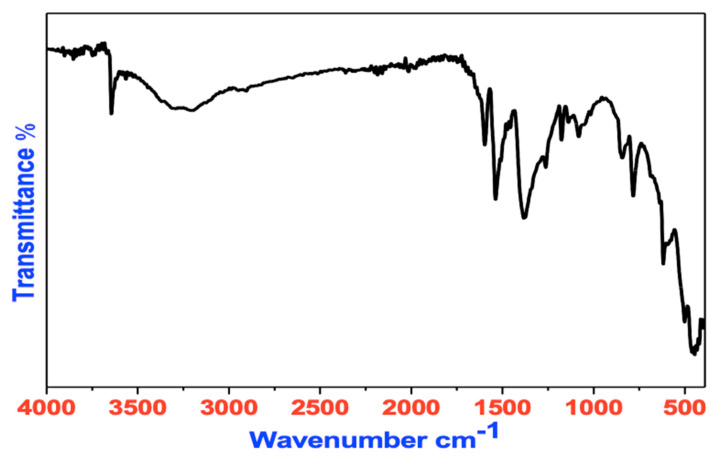
FT-IR of Ni(II) complex.

The principal “infrared spectra” of the fabricated Ni(II) complex are illustrated in Table 2. When the FT-IR spectra of the Ni(II) complex were compared to those of the free PVA and P-ABA ligands, interesting features relating to metal–ligand (M-L) interactions were observed. Based on the FT-IR spectrum, a wide broadband characteristic of the (O-H) stretch vibration of PVA is seen with less intensity of functional vibration groups in the matrix, reflecting the strong interaction between the Ni(II) and PVA ligand. We observed that the ν(O-H) band, which appears at 3400 cm^−1^ in the spectrum of PVA, is shifted to a lower wave number of 3292–3250 cm^−1^, indicating that this group is shared with the metal ions during bonding. The stretching frequency of ν(CH_2_) is seen at 2890–2930 cm^−1^ (a slight shift compared to the PVA spectrum at 2905 cm^−1^). The peaks of ν(C-OC) stretch vibration were listed at 1150 cm^−1^ in the ligand spectrum and 1176 cm^−1^ for the complex [27]. The stretching frequency of ν(NH_2_) was observed at 3234 cm^−1^ for P-ABA as a free ligand, while this value was shifted to 3243–3205 cm^−1^ after complexation [28]. The ν(C=O) stretch vibration is noticed as a medium intensity strip at 1680 cm^−1^ of P-ABA; almost no shift occurs in the spectrum for complex, and the previous ligand cross-linked to metal by NH_2_ goes back to Figure 3. The tape characteristic of the ν(C=N) stretch vibration was recorded at 1640 cm^−1^ for P-ABA (a slight shift compared to the ligand spectrum) and 1630 cm^−1^, and the para substitution is present at 750 cm^−1^ [28]. Briefly, through oxygen atoms, PVA coordinates with metal ions in bidentate mode, while P-ABA operates in monodentate mode through nitrogen atoms for all metal ions. A broad, moderately intense band at 3388 cm^−1^ was assigned to ν(OH) of (H_2_O) for the complex [29,30]. Moreover, the appearance of the absorption bands that are representative characteristic absorption peaks at 550 cm^−1^ and 440 cm^−1^ correspond to ν(Ni-O) and ν(Ni-N), respectively [31,32].

### 3.2. Electronic Spectra and Magnetic Moments

**Figure 4:**
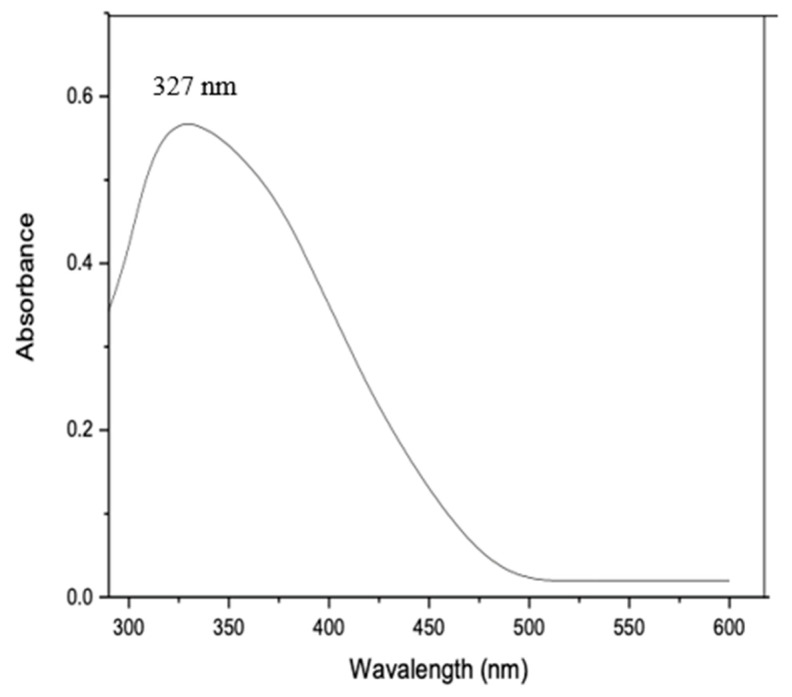
UV-vis spectra of NiO nanoparticles.

A material can either emit or absorb radiation as a function of one particular wavelength. The sample can turn energy from the incident light by the following mechanism: HOMO-LUMO excitation of electrons. In this case, the technique is applied in order to assess the position of the SPR absorption band of metal oxide nanoparticles. In the wavelength range of 300–800 nm, the optical absorption coefficient was calculated for PVA, P-AMB (DMSO, 5 × 10^−5^ M), and their Ni(II) ion, as shown in Table 3 below, and Figure 4 shows the UV-vis spectra of NiO nanoparticles. Absorption peaks were found at 41,841–35,971 cm^−1^ [33,34], which can be attributed to the π-π*, n-π* transition of the PVA and P-ABA. Figure 4 displays the UV-vis spectra of the fabricated nickel oxide, as shown below [35]. The Ni(II) complex presented two absorption bands “typical of 6-coordinate high-spin and low-spin octahedral geometries. These were recorded as 3A_2g_→3T_2g_ and 1B_2g_→1B_1g_ transitions, respectively”, while the moment was 2.59 B.M for Ni(II) complex [36].

### 3.3. Thermal Analysis DTG & DTA

To establish the formation of the metal oxide nanoparticles through the thermal decomposition of the complex and to establish the thermal behavior and crystalline conditions of the complex, differential thermal analysis (DTA) and thermal gravimetric analysis (TGA) were performed. To determine the thermal decomposition, analysis was carried out in the range of 50 to 550 °C under an open atmosphere with a heating rate of 10 °C/min.

#### 3.3.1. Thermal Decomposition of [Ni(PVA)(P-ABA)(H_2_O)_3_]·H_2_O (In Dynamic Air)

**Figure 5:**
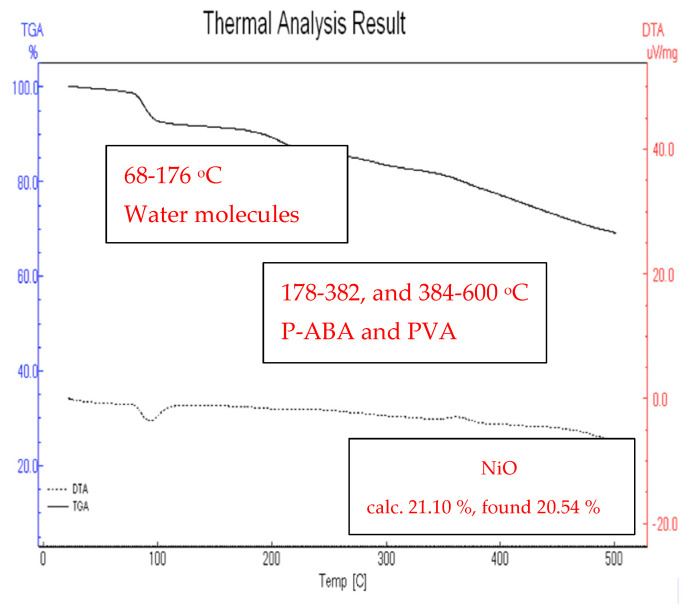
Thermal decomposition of Ni(II) complex.

**Scheme 2:**
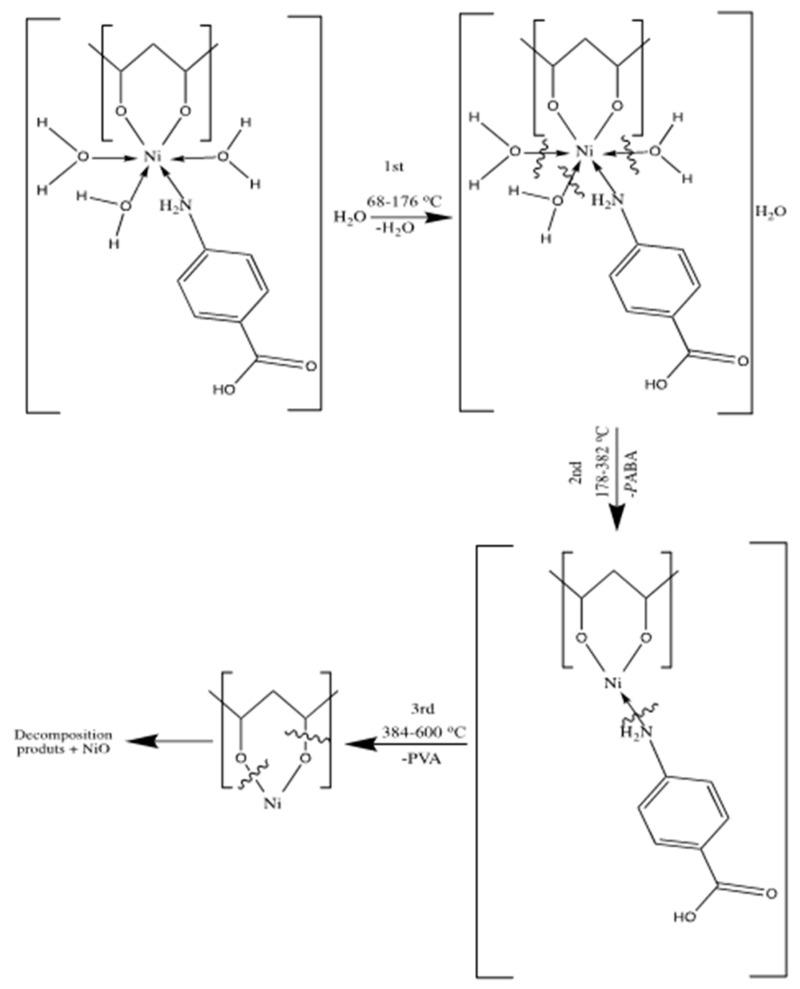
Thermal decomposition of Ni(II) complex.

By examining the thermal curves in Figure 5 of the complex, three steps are observed at 68–176, 178–382, and 384–600, as described in Scheme 2. For the first mass loss (calculated as 20.37% and measured as 19.12%), which corresponds to the loss of water molecules, a DTG peak at 97 °C and an endothermic peak at 99 °C are noticed in the DTA trace. The second and third mass losses are highly correlated with the release of the decomposition of the complex. Two DTG peaks at 350 and 398 °C and a broad endotherm effect at 352 and 400 °C in the “DTA trace” are noticed. The destruction of the complex seems to follow a complicated course, and the residue corresponds to a pure nickel oxide (calculated as 21.10% and measured as 20.54%).

#### 3.3.2. Kinetic Analysis

**Figure 6:**
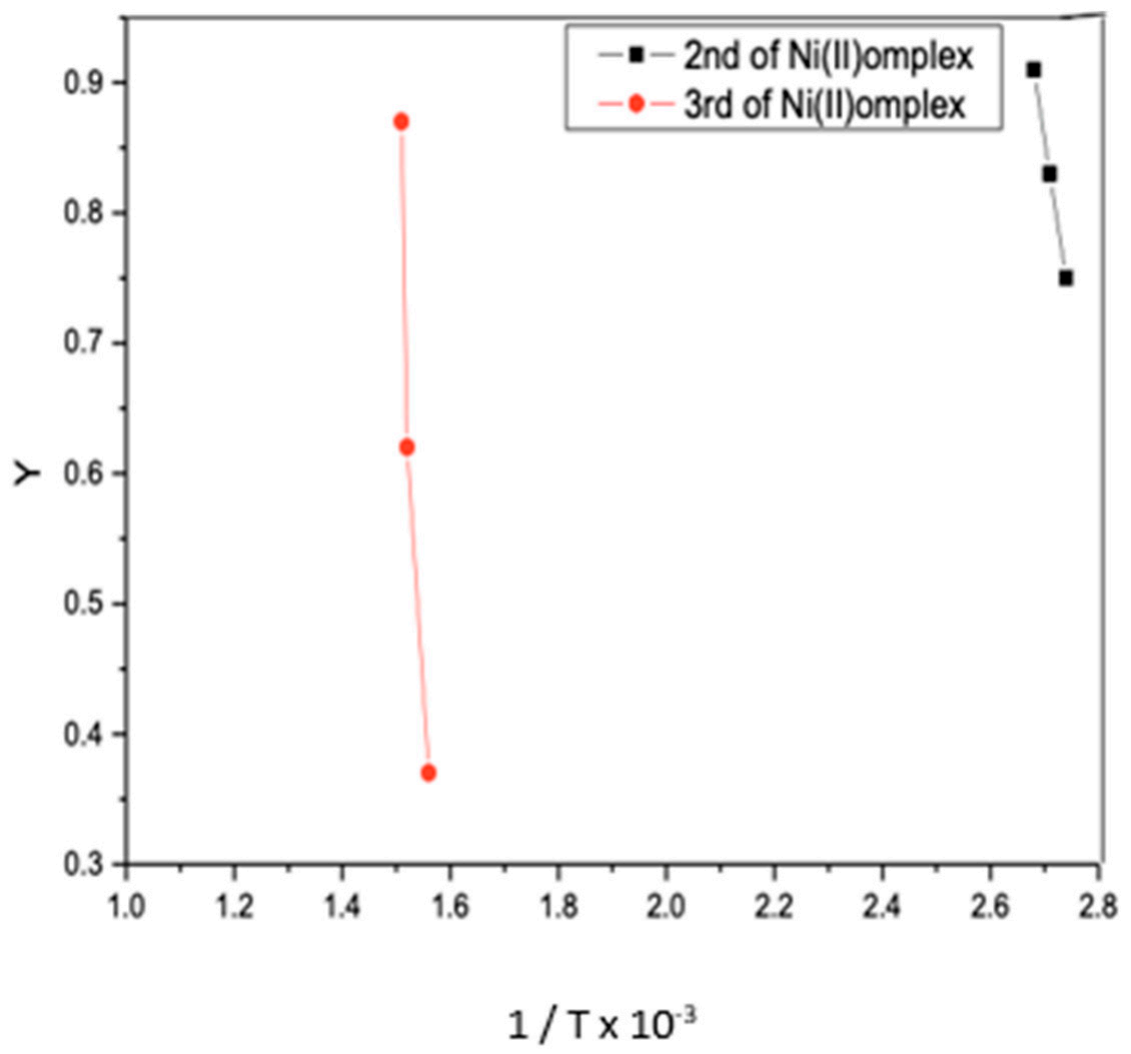
Coats–Redfern of Ni(II)complex.

**Figure 7:**
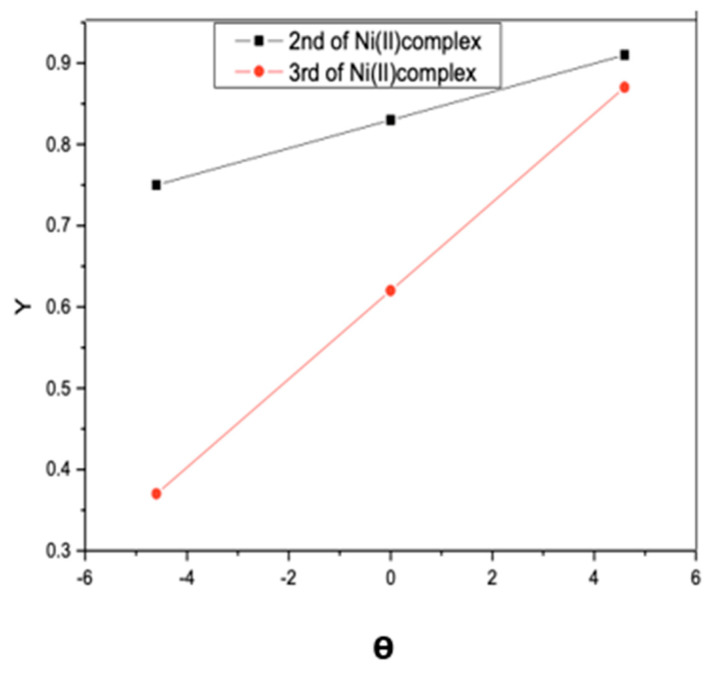
Horowitz–Metzger of Ni(II)complex.

Non-isothermal kinetic analyses of the complexes’ thermal decomposition were performed using two distinct procedures, namely, the Coats–Redfern [37] and Horowitz–Metzger [38] methods, as shown in Figure 6 and Figure 7. The kinetic parameters were evaluated only for stages that were lucid and nonoverlapping. Kinetic studies were not conducted for the decomposition steps, which occur within a quite narrow temperature ambit, resulting in an abnormally steep TG curve for the collection of sufficient data. Moreover, some decomposition steps are too small, such that a sufficient number of points from the TG curve may not be acquired to derive express values for the kinetic parameters within the accuracy of the measurements. The kinetic parameters n, E, and Z were determined using the two preceding methods. When the two sets of kinetic parameters are compared, the difference between them is not as large. The kinetic parameters were calculated and are listed in Table 4.

#### 3.3.3. Thermodynamic Parameters of Ni(II) Complex

The activation variables (∆H*, ∆S*, and ∆G*) for the decomposition stages of the mentioned complex are shown in Table 5, where a negative ∆S* number of stages for the decomposition of Ni(II) complex is put forward, indicating that the stimulated compound is more highly ordered than the reactants, and the reactions are lower than normal. Because the positive number of (∆G*) refers to the decomposition reaction, this is not a spontaneous reaction.

#### 3.3.4. The Decomposition Rate and Stability of the Ni(II) Complex

**Figure 8:**
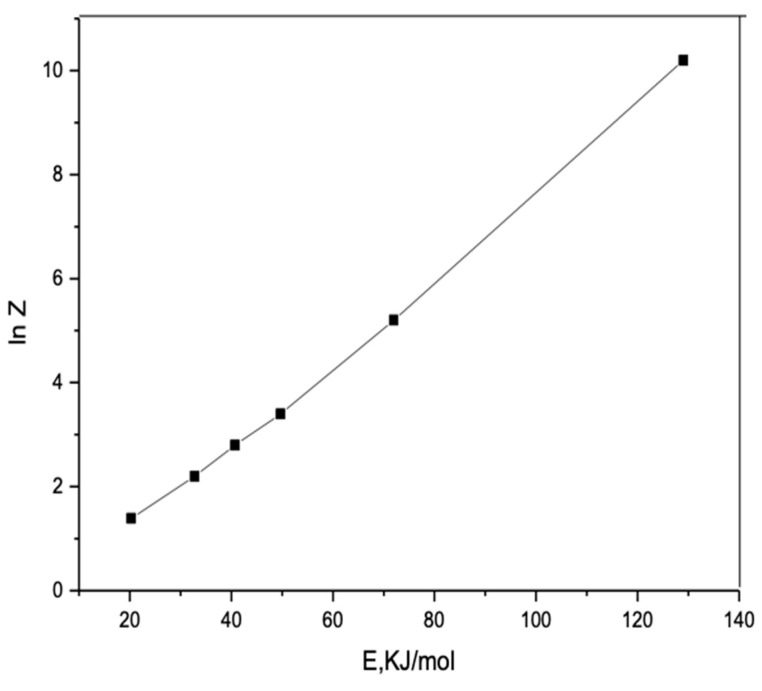
Kinetic compensation effect of Ni(II) complex.

The rate of decomposition of the complex in dynamic air has been deduced from the figures obtained by plotting the fraction decomposition against the temperature of decomposition for the second step, as shown in Figure 6, Figure 7 and Figure 8. Using the characteristic temperature of maximum decomposition rate (DTG, maximum temperature), it is possible to rank the stabilities of the complex (the decomposition rate is determined depending on the inflection point and the “stability of the initial temperature of the first step of the anhydrous” of the compound).

#### 3.3.5. Kinetic Compensation Effect

The kinetic compensation effect (KCE) explains that although the increase of the activation energy is expected to reduce the rate, this change does not reflect the specific set of reactions. As mentioned in many of the non-isothermal methods, KCE was observed here to be inapplicable. It was listed that for the particular process, the value of energy (E) conforms to a straight line with In Z, as shown in Figure 8, obtained by the linear square method for the above complex, which is written as follows:

ln Z = *a*E + *b* (1)

where ***a*** and ***b*** are constants.

- General discussion on the thermal behavior data

The studied complex has some common features in its thermal decomposition. These can be summarized as follows:

The procedure of thermal decomposition of the complex resembles the following thermal decomposition:

A(s) → B(s) + C(g), which can be studied kinetically.

The thermal decomposition of the Ni(II) complex is not easy, and the processes in many cases involve overlapping steps, which, together with the great diversity of possible intermediates, preclude the interpretation of the TG thermograms in detail. However, in some cases, the mass losses can be correlated with the theoretical mass losses.

In most cases, the kinetic parameters observed with the Horowitz–Metzger equation are higher than those obtained from the Coats–Redfern equation. This is due to the inherent error involved in the approximation method employed in the derivation of the Horowitz–Metzger equation.

The order n of the decomposition reactions does not provide any meaningful information about the decomposition mechanisms of the complex. Some of the reaction orders are higher than 1, also implying that thermal decomposition is not a simple process.

The plots of α (fraction decomposed) against the temperature of decomposition provide valuable information about the decomposition rate and stability of the complex.

### 3.4. X-Ray Powder Diffraction (XRD)

**Figure 9:**
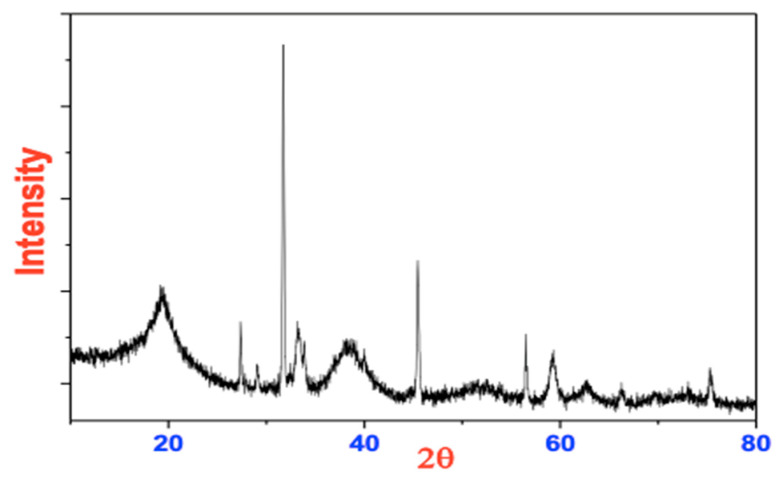
XRD of [Ni(PVA)(P-ABA)(H_2_O)_3_]·H_2_O complex.

**Figure 10:**
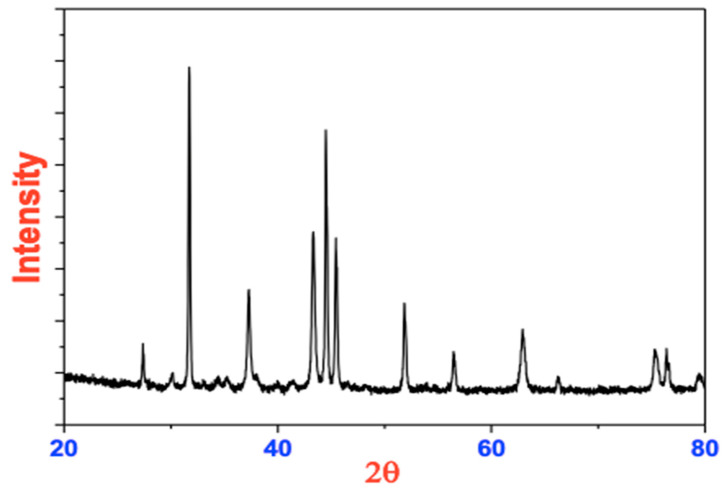
XRD of NiO Nanoparticles.

The XRD of chemically synthesized nickel oxide nanoparticles, as shown in Figure 9 and Figure 10, were obtained “between the 2θ region starting from 20° to 80°”. The figures reveal crystallographic structures, whereas the atoms are arranged in an octahedral shape. Table 6 depicts the diffraction patterns of NiO nanoparticles at a calcinated temperature of 450 °C. These XRD spectra of the metal oxide indicate the number of prominent diffraction peaks at particular angles. The crystallite size increases with increases in peak intensity, which is directly proportional and inversely proportional to the full width at half maxima (FWHM). The protruding peaks were used to calculate the grain size via the Scherrer equation expressed as follows:

D = (094 λ)/(β cosθ)(2)

where “D is the average crystallite size in Å, λ is the wavelength of the X-ray source (1.540) used in XRD, β denotes FWHM (full width at half maximum of the diffraction peak), K is the Scherrer constant (ranges from 0.9 to 1), and θ is the Bragg angle”. The recorded patterns of XRD were in accordance with previous studies, closely matching the reference patterns “(Joint Committee for Powder Diffraction Studies (JCPDS))”. The sharp XRD peaks indicate that the particles were polycrystalline and that the nanostructure formed randomly [39]. The phase peaks of NiO nanoparticles are typically indexed to a hexagonal crystal structure, and the Ni(II) complex is a cubic crystal structure.

### 3.5. Electron Microscopy (SEM & TEM)

**Figure 11:**
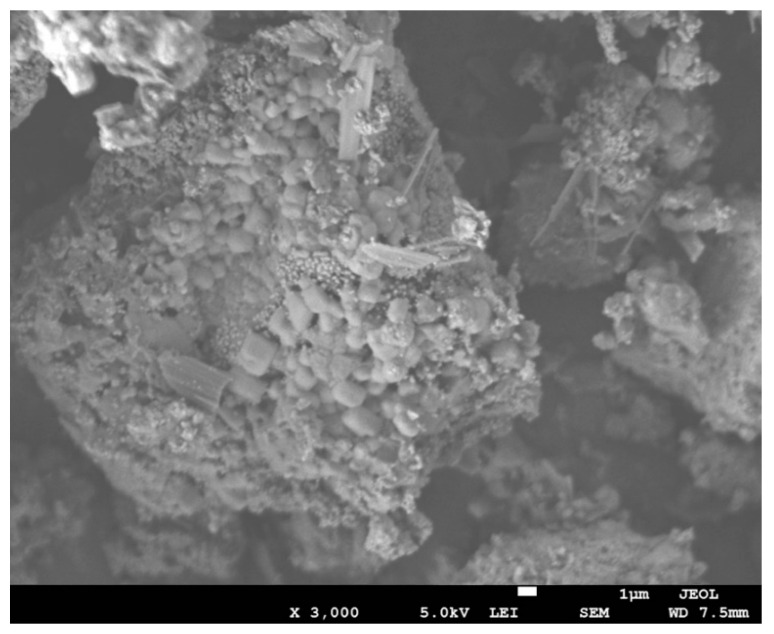
SEM of NiO nanoparticles.

**Figure 12:**
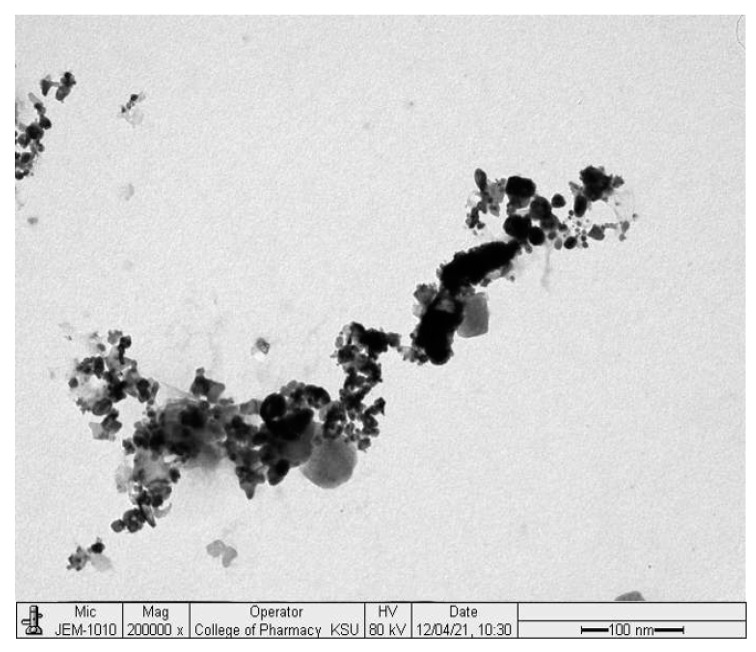
TEM image of the NiO nanoparticles.

The morphological properties and microstructure of the synthesized NiO were observed in SEM and TEM, as shown in Figure 11 and Figure 12. From the images, it is obvious that the particles were highly agglomerated in their natural state. There are some larger particles because of the aggregation or overlapping of small particles. The SEM image displays more agglomeration of nanoparticles with homogeneous morphology. The SEM images of the as-prepared coral-like nickel oxide nanoparticles are illustrated in Figure 11. In addition to the individual particles, some aggregated particles also exist. The as-synthesized metal oxide nanoparticles examined by TEM show spherical structures of NiO nanoparticles; the majority of nanoparticles are sphere-shaped.

### 3.6. Antimicrobial Activity

Using a diffusion method against three strains of Gram-positive cocci (staphylococcus aureus, staphylococcus epidermidis, and *Enterococcus faecalis*), two strains of Gram-negative bacilli (*Escherichia coli* and *Pseudomonas aeruginosa*), one stain of yeast-like fungi (*Candida albicans*), and two molds (*Aspergillus fumigatus* and *Aspergillus flavus*), the antimicrobial activity of NiO nanoparticles was evaluated. The data in Table 7 show that the NiO NPs have extremely good activity versus bacterial strains and fungal strains. In this study, the inhibition of bacterial and fungal growth by the mentioned nanoparticles is discussed as follows. The cell wall of Gram-positive bacteria is composed of “peptidoglycan” molecules. Gram-positive bacteria have a thick wall. Because of the negative charge of peptidoglycans, they bind positively charged ions released by metal NPs in the liquid growth medium. Although “Gram-negative bacteria such as E. coli” allow for a greater number of positive ions to reach the plasma membrane, they are “generally considered less susceptible to antibiotics and antibacterial agents than Gram-positive bacteria” [40].

In order to exercise antibacterial function, NPs should be in direct contact with bacterial cells. The shapes of contact that are accepted include electrostatic attraction, Van der Waal, receptor–ligand, and hydrophobic [41,42,43,44]. The NPs then cross the bacterial membrane and accumulate at the metabolic pathway, altering the structure and functioning of the cell membrane. Subsequently, they are involved with the constituents of the bacterial cell, such as DNA, lysosomes, ribosomes, and enzymes; the effects include oxidative stress, heterogeneous alterations in cell membrane permeability, electrolyte balance disorders, inhibition of enzymes, deactivation of proteins, as well as changes in genetic expression [45].

**Figure 13:**
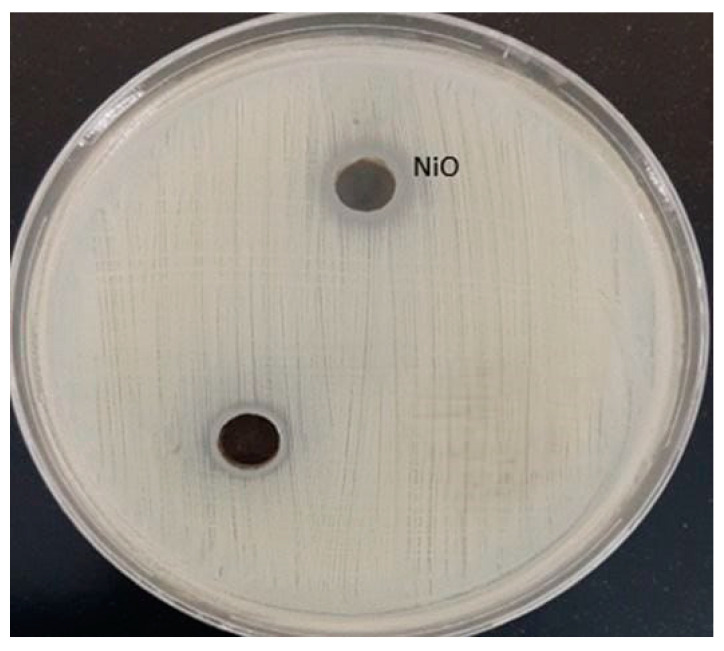
Antimicrobial efficiency of NiO against *staphylococcus aureus*.

**Figure 14:**
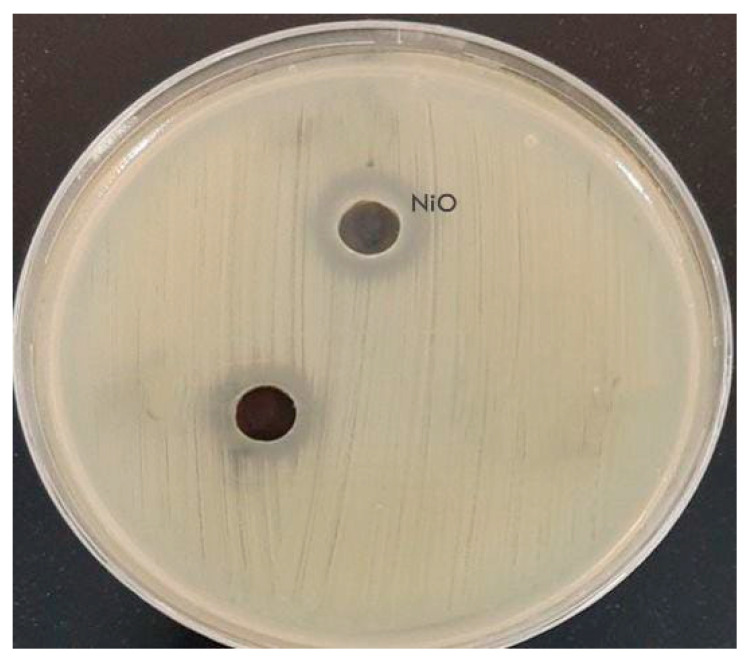
Antimicrobial efficiency of NiO against *Escherichia coli*.

At the end of the analysis, one may conclude that the “Gram-negative bacterial strains” are more effective in relation to the antimicrobial activities than the “Gram-positive bacteria”, as considered from the point of view of its membrane structure. Because of these opposite charges, whereby the reduction of the bacterial cell wall is achieved, the main mechanism of action of metal oxide NPs, in this case, is adhesion. While the two groups may resemble each other in many ways, they differ in some important properties of the membrane structures; the major difference is in the size of the peptidoglycan layer. The accumulation of the metal ions on the surface destroys the cell wall, and the ions diffuse into the cells. This route causes harm to the cell wall or membrane. Figure 13 and Figure 14 illustrate the zone of inhibition for Gram-positive bacteria staphylococcus aureus and the zone of inhibition for *E-coli*-negative bacteria using prepared NiO nanoparticles. The current work demonstrated that the prepared NPs killed bacteria against both Gram-positive and Gram-negative strains.

## 4. Conclusions

In summary, nickel(II) mixed-ligand compounds derived from PVA and P-ABA could be prepared and structurally characterized. We successfully prepared the nickel oxide nanoparticles by calcination of this complex. NiO nanoparticles were successfully synthesized by the calcination method in an oven in the presence of air at 450 °C. The calcination process can effectively remove residues and lead to the better crystallization of nanoparticles, lower cost, and higher-purity nanosized products. Furthermore, it can be scaled to the industrial level. The synthesized NiO NPs were characterized by optical, structural, and morphological characterizations. A hexagonal structure was formed in the NiO nanoparticles. Using UV-vis FT-IR spectra, different functional groups were studied. The SEM and TEM images were used to demonstrate the morphology and the particles’ size, which showed that the NiO NPs prepared by the calcination method exhibited uniform morphology and good particle size distribution. The SEM images display more agglomeration of nanoparticles with homogeneous morphology. Most of the TEM images indicate that the synthesized nanoparticles are spherical in shape. The synthesis of the discussed NPs demonstrated excellent antibacterial and antifungal activities. The advantage of preparing them is explained by the point that, because of their outstanding antimicrobial activity, they can be efficient substitutes in the development of smart systems used for the identification and therapy of pathogenic infections.

## Figures and Tables

**Table 1:** Color, elemental analysis, and decomposition point of the Ni(II) complex.

Compound	MF (M.Wt)	Color	Found (Calcd. %)			mp (°C) (Decom.)	Λ_m_ (Scm^2^ mol^−1)^
			C	H	N		
**[Ni(PVA)(*P*-ABA)(H_2_O)_3_]·H_2_O**	C_10_H_19_N_2_NiO_8_ (353.89)	Light green	34.1 33.93	5.74 5.41	8.86 7.91	276	92

**Table 2:** FT-IR patterns of pure PVA, *P*-ABA, and Ni(II) complex.

Assignment	PVA	*P*-ABA	Ni(II) Complex
**O-H**	3400	-	3260
**O-H** **water**	-	-	3388
**N-H_2_**	-	3234	3209
**C=N**	-	1640	1630
**C-OC**	1150	-	1176
**M-O**	-	-	550
**M-N**	-	-	440

**Table 3:** The UV-vis data and magnetic moments of the Ni(II) complex.

	Wave Number (cm^−1^)	Type of Transition	M_eff_ B.M
**Ni(II) complex**	15,740	d-d transition	2.59
	36,630	n→π*	
	40,485	π→π*	

**Table 4:** Kinetic parameters for the thermal decomposition of nickel(II) complex.

Step	Coats–Redfern				Horowitz–Metzger			
	n	*r*	E	Z × 10^2^	n	*r*	E	Z × 10^2^
**Second**	0.00	0.9918	20.35	1.39	0.00	0.9830	24.36	6.86
	0.33	0.9972	32.88	2.26	0.33	0.9895	35.59	5.14
	0.50	0.9990	40.71	2.82	0.50	0.9962	40.41	1.39
	0.66	0.9994	49.71	3.45	0.66	0.9974	50.52	1.12
	1.00	0.9996	72.02	5.26	1.00	0.9976	70.73	3.07
	2.00	1.000	129.09	10.28	2.00	0.9996	120.13	7.68
**Third**	0.00	0.9918	172.88	1.18	0.00	0.9830	171.32	5.53
	0.33	0.9972	190.26	1.31	0.33	0.9895	192.87	4.47
	0.50	0.9990	211.40	1.46	0.50	0.9962	208.41	6.53
	0.66	0.9994	237.71	1.66	0.66	0.9974	237.70	7.47
	1.00	0.9996	241.94	1.76	1.00	0.9976	239.85	3.14
	2.00	1.000	245.39	1.95	2.00	0.9996	244.80	5.28

**Table 5:** Thermodynamic parameters of Ni(II) compound.

Step	∆S*	∆H*	∆G*
**Second**	−241.98	21.3	110.42
	−248.54	32.53	124.07
	−250.29	37.35	129.53
	−250.64	47.46	139.77
	−250.72	67.67	160.01
	−250.95	117.07	209.49
**Third**	−250.80	21.3	110.42
	−251.13	32.53	124.07
	−251.25	37.35	129.53
	−251.54	47.46	139.77
	−251.90	67.67	160.01
	−251.09	117.07	209.49

∆H*, ∆G* in Kj mol^−1^ and ∆S* in Kj mol^−1^ K^−1.^

**Table 6:** X-ray powder diffraction crystal data of the Ni(II) complex and NiO nanoparticles.

Parameters	Ni(II) Complex	Nickel Oxide (NPS)
**Empirical formula**	C_10_H_19_N_2_NiO_8_	NiO
**Formula weight**	353.89	74.68
**Crystal system**	Cubic	Hexagonal
**a (Å)**	7.97	2.95
**b (Å)**	7.97	2.95
**c (Å)**	7.97	7.24
**α (°)**	90	90
**β (°)**	90	90
**γ (°)**	90	120
**Volume of unit cell (Å3)**	507.35	54.86
**Particle size (nm)**	-	36

**Table 7:** Inhibition zones of NiO NPs.

Tested Organism	NiO
*Staphylococcus aureus*	12
*Staphylococcus epidermidis*	11
*Enterococcus faecalis*	10
*Escherichia coli*	14
*Pseudomonas aeruginosa*	12
*Candida albicans*	16
*Aspergillus fumigatus*	15
*Aspergillus flavus*	12

## Data Availability

The data that support the findings of this study are available from the corresponding author upon reasonable request.
